# The Multi-hub Academic Conference: Global, Inclusive, Culturally Diverse, Creative, Sustainable

**DOI:** 10.3389/frma.2021.699782

**Published:** 2021-07-23

**Authors:** Richard Parncutt, PerMagnus Lindborg, Nils Meyer-Kahlen, Renee Timmers

**Affiliations:** ^1^ Centre for Systematic Musicology, University of Graz, Graz, Austria; ^2^ School of Creative Media, City University of Hong Kong, Hong Kong, China; ^3^ Department of Signal Processing and Acoustics, Aalto University, Espoo, Finland; ^4^ Department of Music, University of Sheffield, Sheffield, United Kingdom

**Keywords:** conference, multi-hub, semi-virtual, hybrid, emissions, inclusion, climate change

## Abstract

New conference formats are emerging in response to COVID-19 and climate change. *Virtual conferences* are sustainable and inclusive regardless of participant mobility (financial means, caring commitments, disability), but lack face-to-face contact. *Hybrid conferences* (physical meetings with additional virtual presentations) tend to discriminate against non-fliers and encourage unsustainable flying. *Multi-hub conferences* mix real and virtual interactions during talks and social breaks and are distributed across nominally equal hubs. We propose a global multi-hub solution in which all hubs interact daily in real time with all other hubs in parallel sessions by internet videoconferencing. Conference sessions are confined to three equally-spaced 4-h UTC timeslots. Local programs comprise morning and afternoon/evening sessions (recordings from night sessions can be watched later). Three *reference hubs* are located exactly 8 h apart; additional hubs are within 2 h and their programs are aligned with the closest reference hub. The conference experience at each hub depends on the number of local participants and the time difference to the nearest reference. Participants are motivated to travel to the nearest hub. Mobility-based discrimination is minimized. Lower costs facilitate diversity, equity, and inclusion. Academic quality, creativity, enjoyment, and low-carbon sustainability are simultaneously promoted.

## Introduction

In the public imagination, brilliant academic research is done by lone geniuses working late among dusty books or in stuffy laboratories. In fact, scholarship and research have always been social enterprises (Montuori and Purser, 1995). In both humanities and sciences, new knowledge is created, and new insights revealed, when experts with complementary backgrounds get together to solve big problems. An important place where people bounce original and promising ideas off each other is the international conference.

Until a few years ago, academics thought nothing of flying from anywhere in the world to a single location and enjoying a few days of intense communication. We returned home with new ideas, plans, and sense of purpose. In 2021, after a year of virtual communication triggered by the COVID-19 epidemic, many are longing to go “back to normal.” That is hardly likely, given the worsening global climate situation. Whereas COVID-19 happened more suddenly, climate change is more important in the long term.

### The Climate Crisis

The global climate crisis is a matter of life and death for a billion people, mostly in developing countries (Chalabi and Foss, 2020; Fong, 2020; Gonzalez-Perez and Neuerburg, 2019; Goshua et al., 2021; Guterres, 2018; Lemery et al., 2021; McMichael, 2017; Parncutt, 2019; Watts et al., 2019). The global situation will almost certainly get steadily worse in coming decades, as global mean surface temperature gradually rises (IPCC, 2018). Even if global net zero is reached, global mean surface temperature will continue to rise, possibly for decades (Sanderson, 2020). Positive feedbacks could push planetary climate across a threshold toward a “hothouse Earth,” in which human survival is unlikely (Steffen et al., 2018).

Whereas aviation contributes about 2.5% to anthropogenic CO_2_ emissions (Lee et al., 2021; Ritchie, 2020), the total contribution of aviation to climate change is about 3.5% (Lee et al., 2021); “aviation emissions are currently warming the climate at approximately three times the rate of that associated with aviation CO_2_ emissions alone” (Lee et al., 2021, abstract; *see* Jungbluth and Meili, 2019). Before the COVID-19 pandemic, aviation was growing globally at about 5% per year (Freeman et al., 2018). If that growth continues at the same time as the remaining carbon budget for 2°C of global warming is consumed, by 2050 emissions from aviation could approach 100% of the remaining carbon budget (Bows et al., 2008). Strategies to stop or reverse the growth include taxing flying, penalizing frequent flyers, abolishing subsidies, and promoting more efficient, slower aircraft (Gössling et al., 2020).

On average, climate change will affect poor countries more than rich countries, although rich countries produce more emissions. Ethnomusicologist Catherine Grant (2018, p. 126) commented that

impacts of climate change are anticipated to be particularly damaging in poorer areas of the world, areas that have least contributed to the problem of climate change (…) and that have fewest resources to cope with it (…) These areas are home to peoples and cultures with which the discipline of ethnomusicology has historically been most concerned. (…) Yet any discussion of climate change in relation to academic activity in general, or academic flying in particular, has always been, and remains, both minimal and peripheral to our thinking as music researchers. Surely the principle of climate justice should feature no less in our research ethics than those principles of inclusion, respect, and mutuality that lie at the very core of contemporary ethnomusicological approaches to scholarship?

Whereas the general public increasingly understands that global mean surface temperature depends primarily on global greenhouse gas concentrations, which in turn depend primarily on anthropogenic emissions, most underestimate the risk (Ballew et al., 2019). Few realize that *total global* emissions must fall significantly in the *next few years*, a goal that in practice can only be achieved if emissions *in every sector* fall significantly and urgently. The complex ways in which humanity as a whole has been dragging its feet for the past few decades suggest that many well-informed and well-meaning people are having trouble converting their understanding of this point into corresponding action. Visible green behavior can help reduce the *value-action gap* (Babutsidze and Chai, 2018); people need to be shown what to do, and how (Omrcen et al., 2018). Another approach is climate change communication that anthropomorphizes nature (Tam, 2014) – explaining that the atmosphere “doesn’t negotiate” and “doesn’t care where the emissions come from.”

In this regard, academics can be important role models. As humanity gradually gets global emissions under control – as it inevitably must – academics in all disciplines, with their unusual social influence and educational privilege, have a special responsibility to play a leading role in the transition (Parncutt and Seither-Preisler, 2019; Roos and Hoffart, 2021).

### Academic Contributions to Climate Change

Internationally mobile academics have high carbon footprints (Middleton, 2019). A long-haul return flight in economy class corresponds to roughly a tonne of burned fossil carbon (creating 3.7 tonnes CO_2_). That figure, which does not consider other greenhouse gases (atmosfair.de), is comparable with driving a car for a year (consuming 30 L of gasoline, or 20 kg carbon, per week) or eating beef for 3 yr (500 g per week, 50 kg CO_2_ per kg beef). “For many academics, the carbon emissions associated with air travel dominate personal carbon budgets, dwarfing other contributions such as from driving or eating a meat-based diet” (Quinton, 2020). It follows that considerations of research ethics, as commonly discussed by ethics committees, might usefully include questions about conference organization (Parncutt and Seither-Preisler, 2019, 2021).

Of all the CO_2_-equivalent created by academic conferences, by far the greatest contribution is from flying – typically, one tonne of carbon for intercontinental participants. Consider air conditioning for a 4-day conference for 400 people in a hot, humid location. The air conditioners might consume 30 kW of electricity for 60 h, total 1800 kWh. For electricity from fossil fuels, 0.5–1 kg of CO_2_ is emitted per kWh. To convert mass of CO_2_ to mass of carbon, divide by 3.7. The corresponding mass of carbon is roughly 500 kg – less than the emissions from flying of one participant. In an order-of-magnitude estimate, air conditioning represents less than 1% of total conference emissions. If electricity is from sustainable sources (e.g. solar panels on the roof), emissions from air conditioning approach zero. Similar rough calculations can be made to estimate contributions from eating meat, disposing of plastic, or watching videos. Beef for the described conference might altogether represent 50–100 kg carbon, disposable plastic 5–10 kg carbon, and audiovisual internet communication 20–50 kg carbon (music-psychology-conference2018.uni-graz.at/en/aims/future). These very approximate calculations confirm that flying is by far the main source of emissions from typical conventional conferences.

Many universities are now evaluating their carbon emissions, including those from flying. The University of British Columbia, Canada, estimated their emissions from flying to be 63–73% of total university emissions (Wynes and Donner, 2018). For Finnish universities, flying typically makes up about half of total emissions, but there is a lot of variation – from (apparently) as low as 10% (University of Helsinki) to 78% (Hanken School of Economics) (Ahonen et al., 2021, Figure 14). Aalto University (2020) calculated independently that air travel constituted 58% of total university emissions in 2019. Scope 3 emissions, defined as “indirect emissions from procurement, business travel, and commuting,” amounted to 79% of the total emissions of De Montfort University (Ozawa-Meida et al., 2013). “Travel for the purpose of dissemination” contributed 69% of the total carbon footprint of Maine’s Sustainability Solutions Initiative (Waring et al., 2014).

One might argue that aircraft will take off anyway, whether we buy a ticket or not. In fact, reducing demand is arguably the most direct way of influencing markets as an individual. The probability of a plane taking off depends mainly on the number of people who buy tickets. Anderson (2013) remarked:

Jump on a plane and you send a suite of very clear market signals. *Please buy* some more aircraft that will operate for 20-to-30 yr and have a design life of 40 yr. *Please build* some more airports. *Please divert* public transport funds so passengers (and shoppers) can travel to the airport on low-carbon trains or trams. *Please expand* the airport car park for when bags are just too heavy to lug on a tram. *Please keep* producing the black stuff – without it we will have invested billions in an industry dependent on kerosene; lock-in *par excellence*. (italics in original)

Another possible argument is this: If the world is aiming to halve total emissions by 2030 and achieve net zero by 2050, academics should similarly aim to reduce emissions from flying to 50% by 2030. But many sectors will fail to achieve that goal, and some may not achieve any reduction at all, for various reasons. Therefore, every sector should strive to reduce emissions as quickly as possible – in any case, faster than agreed in Paris in 2015. In the longer term, each sector must systematically explore their own possibilities and opportunities.

Each sector has “low-hanging fruit” with which to begin their emissions reductions. While these may be harder to locate in some sectors than others, there is no reason to delay taking advantage of them. Regarding direct individual emissions, for example, measures as reducing vehicle engine idling, reducing standby power use, using efficient light bulbs, adjusting household thermostat settings, decreasing water heater temperature, or maintaining recommended car tire pressure can be implemented immediately (Vandenbergh et al., 2007). Big changes in academic conference culture could be implemented with similar rapidity.

So far, few universities have responded adequately to this challenge. To the authors’ knowledge, most universities are still reimbursing the academic travel expenses of their senior research staff, effectively encouraging them to fly – a form of fossil-fuel subsidy. Glover et al. (2018) divided Australian universities into three categories: “Air Travel Ignorers,” “Recognition without Intervention,” and “Air Travel Substituters,” of which the latter “seek to substitute their air travel with a digital form of mobility, usually video conferencing.” The authors noted that

those universities which seemingly have the most progressive policies for more sustainable air travel tend to rely on narrow technological “fixes” such as video conferencing to act as a substitute for air travel. Such policies assume a smooth and uninterrupted adoption of technologies to replace existing academic practices. However (…) transitions toward sustainable practices require the “unmaking” of existing practices as much as they require innovations toward new practices (Shove, 2012). Therefore, we argue that the policy of air travel “substitution” is unlikely to change air travel practices and reduce carbon emissions significantly. New innovations in ways of collaborating and interacting remotely are also needed.

These issues are of particular importance for younger colleagues. On the one hand, younger colleagues are more likely than older colleagues to be personally and directly affected by climate change. On the other hand, younger colleagues need to present their research and meet leading colleagues personally at conferences, to improve their chances of getting research grants and teaching or research positions.

Under these circumstances, things can hardly return to “normal” after COVID-19. To save what can still be saved of the world’s climate and biodiversity for future generations, emissions in all areas must now be drastically and urgently reduced. If current approaches to virtual conferencing are not up to the task, we must creatively and systematically investigate alternative formats.

The realization that academic flying must be severely curtailed could be a blessing in disguise. According to cliché, necessity is the mother of invention, and that may be especially true when the motivation is prosocial (Grant and Berry, 2011). By motivating academics to rethink the purpose and format of academic conferences, the global climate emergency may inspire the emergence of new conference formats that improve on traditional and current formats, regardless of emissions.

## Theory of Low-carbon, Inclusive, Global Conference Organization

### General Criteria for Academic Conferences

Why do we have academic conferences? What are they for? What do we expect from them? What criteria should an international academic conference fulfill? Consider the following points:

*Academic exchange.* Conferences should offer diverse opportunities for colleagues to exchange knowledge, including informing international experts by presenting current work, discussing academic content in larger and smaller groups, and interacting personally, learning from colleagues and generating new ideas and plans.

*Motivational character.* Conferences should motivate participants to carry out globally leading research in the years following the conference, both individually and in groups. Conferences also motivate participants to give excellent presentations as they look forward to catching up with existing colleagues and meeting new ones or impressing possible future employers. Physical meetings can create a feeling of mutual trust that continues for years afterward, enabling productive collaborative work at a distance.

*Inclusivity.* Conferences should allow all active and respected scholars and researchers in a given field or discipline – of any age or gender and in any country – to get together and exchange ideas (Levitis et al., 2021). To our knowledge, this criterion has never been fulfilled. The total cost of travel, registration, and accommodation for one participant at a conventional international conference typically lies between 1,000 and 2,000 USD. Usually, only colleagues from richer countries can afford to attend, and even within the richer countries, many doctoral students are excluded for financial reasons, depending on the field. Other colleagues are unable to travel due to disability or caring commitments. They tend to be forgotten because they are not present, which perpetuates the problem.

*Environmental sustainability.* The total overall carbon footprint of a conference should be as low as possible, both absolutely and per participant. Given the urgency of the global climate crisis, the aim must be to eliminate carbon footprints almost entirely.

At first glance, these criteria contradict. How can a conference with physical face-to-face contact be open to anyone in the world regardless of financial means? How can emissions be reduced while at the same time improving the conference experience? In the following, we will show that it is possible to simultaneously satisfy these apparently contradictory criteria – at least in part – and move stepwise toward new solutions.

### Systematic Emissions Reduction

A possible response to the global climate emergency is to reduce emissions by small but significant amounts, for example by reserving keynote invitations for colleagues in the same country or choosing a conference location that is relatively close to a majority of expected participants. Stroud and Feeley (2015) commented that

the IBS [International Biogeographical Society] meeting in Germany is predicted to result in average emissions of 2.5 tonnes CO_2_ per person. This is 0.2 tonnes CO_2_ more per person than would be incurred if the meeting were held at an overall optimal location of London, UK.

But 0.2 out of 2.5 tonnes is a small saving, and in this scenario the rights of colleagues from other continents are ignored.

Two main approaches to reducing emissions are currently being tried out in different academic disciplines: the *virtual* conference and the *hybrid* conference. Both have specific advantages and disadvantages.

### Virtual conferences

One advantage of a fully *virtual conference* is the chance to reach all colleagues in the world that have good internet connections, regardless of their financial means or mobility. That includes colleagues in non-wealthy countries or students who cannot afford a regular international conference. It also includes colleagues with caring commitments or disabilities. All these groups may welcome virtual conferences for this reason.

But a virtual conference also means sitting for hours at home or in the office in front of a laptop, often at strange times (depending on international time differences). After a year of COVID-19, people are understandably exhausted (“zoomed out”). Participants at virtual conferences may attend fewer talks and they may be more easily distracted during those talks by whatever is happening where they physically are, as well as new emails or social media.

Although virtual interaction can sometimes promote creativity, for example by allowing people to focus and to suppress tendencies toward social conformity (Kearney 2020; Thompson, 2021), on balance there is no clear difference in creativity between real and virtual groups (Andres, 2002; Bathelt and Turi, 2011). Han et al. (2017), p. 261) found that while some aspects of virtual interaction can inhibit the creativity of groups, they can be made up for in other ways:

This research uncovered distrust, personality differences, generational differences in views, scheduling issues and technology difficulties as the top five inhibitors for virtual team creativity and success. The authors identified seven main strategies for developing virtual team creativity and success. The authors found that building “team norms” and guidelines to encourage positive interactions between team members can facilitate team creativity. In addition, a concept of trust-based open communication was identified as one of the important strategies when teams actively use technology-mediated communication tools.

### Hybrid conferences

The *hybrid conference* is a conference at a central location, to which some participants travel; others (*virtual participants*) stay at home and communicate electronically. Two of the authors organized a conference of this kind in 2019 in Graz, Austria (Conference on Interdisciplinary Musicology). Many such conferences are now being held in different disciplines.

The advantages of a hybrid conference are clear. Like a virtual conference, a hybrid conference is open to anyone in the world, regardless of mobility. If the fee for virtual participation is low, almost anyone can afford it. Therefore, a low fee for virtual participation, especially for participants with low financial means, is an important way to promote inclusion. But hybrid conferences also have significant problems.

At a hybrid conference, physically present participants have a very different experience from virtual participants. Physical attendees meet face to face and mix academic discussion with small talk, as at a traditional single-location conference. The conference format offers them the additional benefit of virtual presentations from colleagues who would not otherwise have been able to attend, and face-to-face local discussions following those talks. For the virtual participants, the entire event involves looking into a laptop or other electronic device. No matter how hard we try to compensate for those disadvantages – for example with advances in virtual meeting technologies and virtual socializing formats – virtual participants at hybrid meetings still experience a disadvantage.

From a sustainability viewpoint, hybrid conferences may motivate colleagues to fly at a time when we should be encouraging people to stay grounded. At the risk of overstating the case, one might compare a hybrid conference with a behavioral experiment in psychology. By a process of operant conditioning, hybrid conferences train participants to fly by providing salient emotional rewards if they do so. The reward (reinforcement) is almost immediate: it happens soon after conference participants disembark and start to meet old friends and conduct interesting conversations. Immediate reinforcement is known to strengthen the conditioning effect (Holland and Skinner, 1961). Of course, things are more complex than that: many participants are aware of the flying is problematic and actively seek alternatives.

Whereas hybrid conferences may be good news for colleagues who can attend such a conference for the first time, they also offer an inferior experience to virtual participants. Like a conventional single-location conference, hybrid conferences systematically discriminate against colleagues with limited finances, caring commitments, or disabilities. The less mobile find themselves stuck on the periphery, while the more mobile are situated near the centre and enjoy the corresponding benefits. The situation can be compared with institutionalized discrimination or even colonialism (Parncutt and Seither-Preisler, 2021).

### The Multi-hub Conference

A multi-hub conference is distributed across several global locations (Coroama et al., 2012; Parncutt et al., 2019). People meet personally at several hubs, spread across the planet. All talks at each hub are shared with all other hubs, either in real time (permitting live questions from the audiences at other hubs) or as video recordings. Social interaction among hubs happens at virtual socializing sessions that also include face-to-face interaction.

The multi-hub format combines aspects of virtual and hybrid formats. The conference is semi-virtual, in two senses. First, it involves face-to-face communication with some participants and virtual communication with others. Second, during the conference participants can repeatedly choose between parallel real and virtual presentations. Beyond that, not all participants travel to hubs; some participate purely virtually.

The aim of this article is to promote and describe the multi-hub option in detail. While not perfectly solving the addressed problems, we believe that the approach has considerable potential. So far, that potential has barely been appreciated, for two reasons. First, the approach challenges both organizers and participants to think very differently about conference logistics. Second, the approach has not yet been fully implemented. Colleagues have not yet experienced and discussed the solution that we consider here to be ideal.

van Ewijk and Hoekman (2020) investigated emission reduction possibilities for global conferences of the International Society for Industrial Ecology. They proposed that 5% of emissions could be saved by avoiding short flights, 4–14% by introducing a carbon tax of 100$/t CO2e, 20–30% by having 10% of attendees (those who travel furthest) attend virtually, 25–50% by splitting the conference across two hubs, 46–75% for three hubs, and 82% for the latter combined with land transport – confirming the emissions-reduction potential of multi-hub conferences.

The multi-hub conference combines aspects of virtual and hybrid conferences. It is like a virtual conference in that all sessions are live-streamed. Like a hybrid conference, it is *semi-virtual,* in two senses. First, it involves face-to-face communication with some participants and virtual communication with others. Second, during the conference participants can repeatedly choose between parallel real and virtual presentations.

The multi-hub option helps colleagues in all parts of the world to establish, promote, organize, or institutionalize their academic discipline locally. Neither virtual nor hybrid conferences have this potential, unless virtual conferences organizers are globally distributed, promoting the participation and visibility of researchers in different regions (as in ICMPC16-ESCOM11 in July 2021). If a single-location conference is moved to an “exotic” location, the advantage for colleagues in that location is offset by the environmental disadvantage: the carbon footprint of such a conference is typically even higher than that of a traditional conference in a rich country – both for the conference as a whole and the average participant (Fasciani and Goode, 2021).

In a multi-hub conference, we propose giving every hub extended and repeated opportunities to communicate, in real time and in normal working hours, with every other hub. That enables joint sessions of various kinds (talks, poster sessions, meetings, workshops, demonstrations). To maximize inter-hub exchange and connectivity, and hence global outreach, each hub can communicate in real time toward the East in the morning (“AM”) sessions and the West in the afternoon/evening (“PM”) sessions, with a long break (“siesta”) in between (*see* Table 1). Each hub can also communicate in real time toward the North or South.

**TABLE 1:** Proposed organization of the global 24-h program.

UTC (hours)	0–2	2–4	8–10	10–12	16–18	18–20
**Tokyo (+9) and Asia-Pacific**	Early AM	Late AM	Early PM	Late PM		
**Los Angeles (−7) and Americas**	Early PM	Late PM			Early AM	Late AM
**London (+1) and Europe/Africa**			Early AM	Late AM	Early PM	Late PM

The times in UTC in the top row are convenient if reference hubs are located in the time zones of London, Tokyo, and Los Angeles in the summer: for example, UTC8 is 9:00 local time in London. The program at each reference hub runs 9–21 local time (break: 13–17) and comprises four slots: *Early AM* (9–11), *Late AM* (11–13), *Early PM* (17–19), and *Late PM* (19–21). At additional hubs, timing is either the same (9–21) or ± 1–2 h (7–19, 8–20, 10–22, or 11–23). The entire plan may be shifted forwards or backwards by a constant number of hours without changing the basic structure.

Some of the content that is missed during the night at each hub can be presented, if desired, as video recordings on the following day. That applies especially to keynotes, of which there can be one at each hub. Beyond that, individual participants can be given access to all presentations in the entire conference as recordings.

At each hub, and at any time during the working day, any number of parallel semi-virtual sessions can be presented. Participants can choose freely among them – like at a hybrid conference. Local presentations have the advantage of physical directness, whereas virtual presentations, given that there are usually more of them (depending on the number of hubs), may be more relevant (researchers elsewhere working on similar topics), more interesting (lesser known), or of higher quality, on average.

Whereas colleagues from all over the world can participate in all three conference formats – virtual, hybrid, and multi-hub – only virtual and multi-hub formats treat all colleagues equally, and only multi-hub and hybrid formats permit a high proportion of face-to-face interaction, similar to a traditional single-location conference experience. Seen in that way, the multi-hub conference may be the best of the three.

### Previous Multi-hub Conferences

Multi-hub conferences may be organized within or near to a single time-zone. An advantage of such a format is that the entire program is confined to normal working hours at all locations. For example, Mercator Research Institute (2021) is planning a Climate Neutrality Forum on 8–10 September 2021 to be conducted simultaneously in Berlin, Milan, and Oxford. If most participants at this event are European, the multi-hub format will significantly reduce emissions from flying. If, however, there is global interest, the format will neither significantly reduce flying nor improve inclusion on a global level.

A multi-hub conference across widely separated time zones was implemented by the two of the authors (RP and NMK) in 2018. At the 15th biennial International Conference on Music Perception and Cognition (ICMPC), which was combined with the 10th triennial ESCOM conference (“ICMPC15/ESCOM10”) there were four hubs: Montreal, La Plata (Argentina), Graz (Austria), and Sydney.

The result was a compromise, in at least two ways. First, the time difference between the Sydney and Montreal hubs was relatively big: 10 h (ignoring the international date line). That caused significant programming problems: for 2 h per day, the Sydney hub was isolated from all other hubs. Previous attempts to convince colleagues of the necessity for a hub on the West coast of North America had unfortunately failed. Second, although the carbon emissions per participant were reduced by roughly 70% (according to calculations carried out independently by Wegener Centre for Climate and Global Change in Graz), many colleagues did indeed fly. We had recommended that colleagues choose surface transport as far as possible, and data collected at registration showed that many did that. But it was not our intention to prevent flying or to enquire about reasons for flying. Rather, our intention was to reduce the overall carbon footprint, increase environmental awareness, and make it easier for any qualified colleague in the world to participate. In any case, colleagues who fly for a different, urgent reason may reasonably combine that trip with a conference, research project, family event, and/or holiday.

Author RT is now organizing ICMPC16/ESCOM11 in 2021 with hubs in Australia, Azerbaijan, Colombia, India, Lithuania, Mexico, Poland, South Africa, and the UK. Due to the pandemic, some hubs may operate fully virtually. Author PML is organizing “Data Art for Climate Action” (DACA) in 2022 with hubs in Hong Kong and Austria.

Fraser et al. (2017), p. 544) proposed a conference format called “multilateral hub and node” that

Incorporates all the design elements of the multihub and node model, but the elements are replicated across multiple time zones. This … addresses the potential time and resources constraints to conference attendance by improving accessibility on a global scale. This model facilitates the possibility of research and leisure in different countries, should delegates choose to travel to an international hub. By having a number of international hubs in different time zones, this model maximizes the possibility of visiting family and affiliated research institutions. However, time differences reduce the possibility of real-time interaction with other hubs and nodes.

Levitis et al. (2021) proposed in the caption to their Fig. 2:

Three time zone hubs for global accessibility: Asia Pacific; Africa, Europe and Middle East; The Americas. … For example, researchers based in New Zealand can attend hub 1 during 14:00–18:00 (local time). They will be joined in this hub by researchers from Bangladesh for whom the content will happen 8:00–12:00 (local time). … The three time zone hubs make is possible to schedule 4 hours of content each day while remaining within typical working hours for nearly all time zones.

A three-hub conference was proposed by Klöwer et al. (2020) for future international conferences of the European Geosciences Union, the Japan Geoscience Union, or the American Geophysical Union. The proposed locations – Chicago, Tokyo and Paris – were chosen to minimize the total emissions of the conference, which the authors estimated would fall by 80%. But their selection of hubs was not ideal, as they themselves noted:

Critics might counter that such a model would still disadvantage academics in parts of the world remote from these hubs. (...) Questions of equity are important, and need more consideration to avoid exacerbating existing inequalities. (…) Participants would have to accept sessions occurring at unconventional hours, but this is likely to be less stressful than back-to-back intercontinental flights.

Regarding the timing of the program, Klöwer et al. (2020) argued that

Sessions with high attendance could be held in each hub in the afternoon, to allow live late-evening and early-riser participation at the other two hubs. Participants would have to accept sessions occurring at unconventional hours, but this is likely to be less stressful than back-to-back intercontinental flights. (p. 358).

In that scenario, the program would be more interesting in Paris than in Chicago or Tokyo. The time difference between Paris and Tokyo is 7 h; between Paris and Chicago, 7 h; and between Tokyo and Chicago, 10 h. It is more difficult for Tokyo and Chicago to communicate with each other in real time for long periods than it is for Paris to communicate with the other two hubs, which could motivate colleagues to fly to Paris.

### Three Reference Hubs, 8 Hours Apart

We propose solving problems arising from existing multi-hub conference formats as follows:

#### Choose Three Reference Hubs Whose Time Zones Are Exactly Equally Spaced, 8 h Apart

Avoid hub constellations in which most hubs are within or close to the same time zone, and those in which three hubs are only approximately equally spaced. Be strict and inflexible about the equal-spacing criterion from the start, because the advantages outweigh the disadvantages. The advantages include:

*Connectivity:* If the program at each additional hub is simultaneous with the program at the temporally nearest reference hub, every hub can communicate with every other hub for four continuous hours each day, and all sessions at all hubs can be simultaneous with sessions at about 2/3 of the other hubs.

*Flexibility:* The conference program is easier to draw up because the temporal boundaries are decided in advance. In particular, it is easier to organize joint sessions that involve several accessible hubs in real-time communication.

*Equality:* Equal spacing of reference hubs puts the hubs on a more equal footing, reducing the chance that one of them will be perceived as central, such that colleagues will be motivated to travel to it rather than to the closest hub.

The options for choosing reference hubs that satisfy this criterion are numerous (*see* Table 2 for examples), and they change from one time of year to another due to Daylight Saving Time (DST).

**TABLE 2:** Possible locations for reference hubs for a hypothetical conference in April 2021.

Time zones	Possible locations	Evaluation
0, 8, 16	Dakar, Beijing, Anchorage	moderate
1, 9, 17	London, Tokyo, Los Angeles	excellent
2, 10, 18	Berlin, Sydney, Phoenix	good
3, 11, 19	Jerusalem, Solomon Islands, Chicago	poor
4, 12, 20	Yerevan, Auckland, New York	moderate
5, 13, 21	Islamabad, Midway Island, Rio de Janeiro	poor
6, 14, 22	Dhaka, Honolulu, Nuuk	poor
7, 15, 23	Jakarta, Adak, Cabo Verde	poor

Time zones are expressed in hours ahead of UTC (*see* timeanddate.com). The “evaluation” column is an educated guess based on the number of potential participants living near the smallest hub or the likelihood of participants traveling to the smallest hub without flying. The table is different in summer and winter and may change from month to month due to variations in DST.

#### Locate all Additional Hubs in Time Zones That Are Within 2 h of Reference Hubs

Allow any suitably qualified and experienced colleague in any part of the world to establish an additional hub whose program is simultaneous with the temporally nearest reference hub, provided the new hub is not too physically close to an existing hub. That colleague should be free to run an independent budget and charge an independent registration fee that corresponds to the cost of living or gross domestic product (GDP) of the country in question. In that way, as the number of global hubs increases and the carbon efficiency of surface transport options improves, the travel-based carbon emissions per participant will approach zero. Criteria for selection of hubs (whether reference hubs or not) include minimizing flying, aiming for hubs of roughly equal size (equal numbers of participants), enabling new colleagues to participate, and choosing different locations for successive meetings.

#### Treat all Hubs (Reference or Not) Equally From the Point of View of Programming and Content

From the perspective of the conference program and the conference experience, all hubs should be as equal as possible. Avoid creating a central hub and adding satellites, like a colonial power with its colonies. Instead, treat conference hubs like independent computer servers that are networked across the internet in a peer-to-peer (P2P) network – a network architecture with equally privileged participants.

#### Avoid Sessions at Unconventional Hours by Limiting Real-Time Communication to Two of the Three Reference Hubs and Their Associated Additional Hubs

At a conference with three equally spaced reference hubs, all sessions can involve two of those hubs plus all additional hubs whose programs are simultaneous with those hubs. Participants at other hubs, for whom those sessions happen at night, can be offered the option of watching and commenting on a video the next day. In that case, an acoustic group discussion is still possible.

Sessions of interest to all participants at all hubs can be split into two parts, the first involving two reference hubs and the second involving the remaining reference hub. In the second part, participants watch and comment on a video recording made a few hours before. For example, a three-hub conference can have three keynote addresses, each of which is experienced in real time at two of the reference hubs (always a different combination) and watched later at the third. Society meetings can be organized similarly.

*Summarizing*, the proposed multi-hub solution appears optimal within the constraints of current technologies. Most general criteria for a good academic conference can be met if:

• the conference is split across several nominally equal hubs;
• the time differences between three of the hubs (*reference hubs*) are exactly 8 h;
• the working time at each hub is divided into two halves, separated by a long break (siesta);
• the second half always begins 8 h after the first; and
• the program at each additional hub is simultaneous with that of the nearest reference hub.

That way, each hub can interact one-to-one with all other hubs in real time, maximizing inter-hub connectivity. Hub organizers can decide whether to offer the entire global program at their hub in parallel sessions (which is always possible in this approach) or to be more selective. In any case, all participants have access to all events, whether live or after a delay, and whether in a conference session or individually.

### Four Reference Hubs, 6 Hours Apart?

The working hours in the three-reference-hub format can be strenuous, especially if shifted forwards or backwards by more than 2 h. What about a solution with four reference hubs separated by 6 h? One possibility in July 2021 (according to timeanddate.com) is Phoenix, Accra, Dhaka, and Auckland. Another is Honolulu, Boston, Frankfurt, and Beijing.

To line up programs across time zones in this case, the afternoon session would start 6 h later than the morning session. The daily schedule at each hub could be shortened to last 9–12 and 15–18 local time. Each reference hub might then communicate with the two neighboring reference hubs in real time, one in the morning and the other in the afternoon – but never with the reference hub on the other side of the world. In this solution, and imagining additional hubs, each hub can communicate with about 3/4 of the other hubs in real time, whereas in the three-hub solution above, every hub can communicate with every other hub.

The logistic advantage of the four-reference-hub approach is the shorter working hours, which make it easier to shift working hours at each hub forward or backward, leading to more flexible solutions. The disadvantage is the inability to communicate with all hubs in real time, which from our perspective is the most important point, and the reason why we favor the three-reference-hub approach.

## Practical Implementation

### Locating Hubs

In the three-reference-hub model, the placement of reference hubs is largely determined by the earth’s largest ocean, the Pacific. Relative to Coordinated Universal Time (UTC), which for the present purpose is the same as Greenwich Mean Time (GMT), Pacific time zones range from UTC+10 h on the East coast of Australia (or UTC+12 in New Zealand) to UTC−8 on the West coast of North America (or UTC−5 on the West coast of South America). To ensure that reference hubs are exactly 8 h apart, two of them must be located near the Pacific rim: in Japan, Eastern Australia, or New Zealand on one side, and the West coast of North or South America on the other. That in turn limits options for the third main hub, which must be in Europe, Africa, or the Middle East.

Many academic disciplines have separate societies in North America, Europe, and Asia. The three geoscientific societies mentioned above are examples. In music cognition (the primary discipline of authors RP and RT), there is the Asia-Pacific Society for the Cognitive Sciences of Music (APSCOM), the Society for Music Perception and Cognition (SMPC) in North America, and the European Society for the Cognitive Sciences of Music (ESCOM). Author PL is a member of the International Computer Music Association, which divides its activities into three regions: Americas, Europe, and Asia-Oceania. A conference with three reference hubs is ideal for such disciplines: each regional society, chapter, or region can be responsible for one reference hub.

Once the reference hubs are established, colleagues can be invited to propose additional hubs at any global location. Additional hubs are equal to reference hubs in every way except for convenience of working hours. The program at each additional hub is simultaneous with the program at the temporally closest reference hub. To avoid very early mornings and very late evenings, additional hubs should be placed in time zones that are within 2 h of reference hubs. If the program runs 9–21 local time at the reference hubs (break: 13–17), it can be 8–20 or 10–22 for hubs that are 1 h away, and 7–19 or 11–23 for hubs that are 2 h away. Locations are that excluded by this criterion can be included when the conference is repeated in a later year by shifting the reference hubs relative to UTC. If the conference is moved from summer to winter or vice-versa, variations in DST can create opportunities for new hub locations.

Additional hubs should be placed to avoid flying from North to South. For example, if there is a reference hub in Korea, Japan, or China, there should be an additional hub in Australia or New Zealand. If there is a reference hub in South Africa, there should be an additional hub in Europe, and similarly for North and South America. That being the case, the minimum number of hubs is six, and a reasonable number of hubs for a larger academic discipline with global outreach might be ten.

Conference organizers may find the following more specific recommendations helpful. We suggest the following procedure:

1. Choose one of the time-zone options listed in Table 2 (e.g., UTC 1, 9, 17). If a multi-hub conference with three equally-spaced reference hubs has already happened in your discipline, choose a new, different option from the table. Alternatively, hold the conference at a different time of year, taking advantages of changes in DST.
2. Systematically list colleagues that are located within the three chosen time zones and have the required qualifications, experience, and resources to organize a hub.
3. Approach the most promising candidates and establish three reference hubs that are 8 h apart.
4. Only after that, start to approach colleagues in other locations for additional hubs.

All hub organizers should fulfill the same criteria. They should be appropriately qualified and experienced according to the standards and traditions of the discipline in question. They should have access to the necessary resources, including rooms and equipment. They should be in a good position to attract a stated minimum number of participants by surface transport.

When approaching potential hub organizers, emphasize the advantages of accepting the invitation without underestimating the work involved. Organizing a conference can have important spinoffs for colleagues working near that location. It can allow them to create new local academic infrastructures, promote new local research, and attract new grant money. Young organizers typically improve their academic profile and, in that way, their chance of getting academic positions and grants.

If at some point in this process there is a risk that one hub will dominate over all other hubs, consider adding a new hub that is close enough to attract many participants that would otherwise have attended the dominant hub – but also far enough away to represent a different geographic area. For example, if there is a strong research group in France, such that the French hub may be perceived as central, the imbalance can be alleviated by adding an extra hub in a neighboring country such as Germany or Spain.

The biggest population centers in a given time zone can be found by consulting an internet source such as mongabay.com “World’s largest cities.” Lists of that kind could enable a preliminary analysis of possible conference locations, evaluating their suitability according to criteria that depend on the academic discipline and the geographic distribution of its qualified representatives. The result will be different for disciplines such as Spanish Literature or Electronic Engineering.

### Program Structure

The task of drawing up a global 24-h program is radically simplified by confining all program content to three equally-spaced 4-h UTC timeslots. To ensure that adjacent reference hubs can work together in real time, AM and PM slots can last 4 h each, with PM slots beginning exactly 8 h after AM slots. Both AM and PM may then be divided into two 2-h slots, as follows:

The first half hour of each 2-h slot can be for *virtual socializing*, equivalent to the traditional coffee break. If all participants are encouraged to participate in virtual socializing, organizers are more likely to find everyone seated in time for the first talk of the subsequent 90-min slot. That slot might comprise three regular talks of 30 min or a keynote of 60 min followed by a discussion of 30 min. Each conference and each academic discipline will divide these slots up in different ways, depending on aims, content, and tradition. A unified time structure across hubs allows participants to change rooms between talks.

Working sessions can be limited to four 2-h slots. At reference hubs and leaving out social sessions, these working times might be 9:30–11:00, 11:30–13:00, 17:30–19:00, and 19:30–21:00 local time. For convenience, these timeslots can be labelled *Early AM, Late AM, Early PM,* and *Late PM*, respectively. The structure is illustrated in Table 1.

### Diversity, Equity, and Inclusion

The format that we are proposing optimally promotes diversity, equity, and inclusion (DEI). With three equally spaced reference hubs plus any number of additional hubs, almost any qualified colleague, anywhere in the world, can participate fully in the conference. Those who can travel to the nearest hub do so; they are treated equally and enjoy the same conference experience, depending only on the organization of their local hub and the number of people attending at that hub. To encourage hub equality, hubs with more financial resources may agree to financially support those with less.

As at a hybrid conference, any number of virtual participants can stay at home due to caring commitments or immobility, or for any other reason. They have virtual access to the entire program in real time. They do not enjoy the same conference experience as those attending a hub physically, but the conference does offer them the maximum possible benefit given the practical limitations.

*New hubs* are hubs in locations that have not previously hosted an international conference in the academic discipline in question. They may or may not be reference hubs. New hubs are opportunities to create new local and regional academic societies and to promote the discipline of the conference at regional universities. That can have interesting implications for other academic disciplines. It can also contribute positively to international development and may even be seen as a form of international aid.

### Examples of Reference Hub Combinations

Table 2 shows possible combinations of reference hubs that are exactly 8 h apart in April 2021. (We wanted to present a solution for July 2022, but at the time of writing there was uncertainty about whether there would be DST in Europe in 2022; Boffey, 2019.) The listed hub combinations were found by exploring the global time zone map at timeanddate.org. The proposed hub locations were arbitrarily chosen from the larger population centers in each time zone.

The best solution listed in Table 2 in terms of proximity to larger population centers is London, Tokyo, and Los Angeles, or other locations within the same time zones. The second-best solution is Berlin, Sydney, and Phoenix. Further possibilities are Dakar, Beijing, and Anchorage; and Yerevan, Auckland, and New York.

The above solutions are for summer in the Northern Hemisphere. At different times of year, additional options (not shown) emerge due to variations in DST. DST is currently applied in most European and North American countries and Australia, but not in most African and Asian countries, and in future it may be abandoned in Europe. Conference organizers can take advantage of this variation, making multi-hub conferences more inclusive of colleagues in all geographic regions by experimenting with different combinations of reference hubs at different times of year. For example, a conference might be held in the northern summer on one occasion and the southern summer on another.

### Examples of Additional Hub Combinations

**FIGURE 1:**
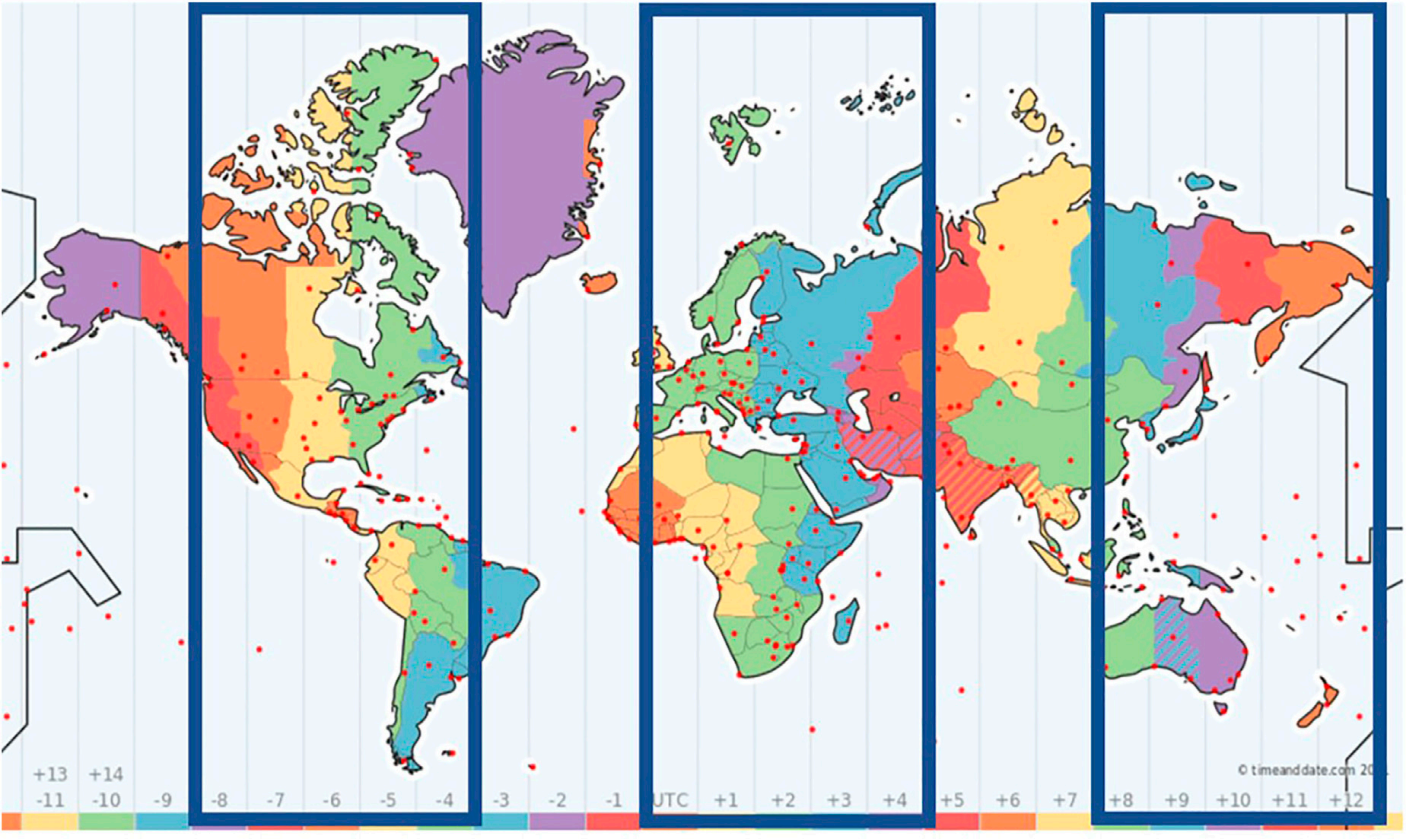
Explanatory sketch showing possible locations for additional hubs in a specific case. The boxes roughly mark the boundaries within which additional hubs could be located if reference hubs are 2, 10, and 18 h ahead of UTC (e.g., Berlin, Sydney, and Phoenix in the summer) and the time difference between reference and additional hubs is limited to ±2 h. The boxes are 5 h wide (−2, −1, 0, 1, 2) and the gaps between them are 3 h wide. Note that boundaries between time zones can deviate markedly from corresponding meridians, and DST is applied inconsistently in different countries. Therefore, the map is for initial guidance only. When considering additional hub locations (roughly 500 cities have more than a million inhabitants; 4,000 have more than 100,000), look up the time zone of each location separately during the period when the conference will be held. Consider also the organizer’s qualification and resources, internet speed, and travel and accommodation options. The map is from timeanddate.com/time/map/, where users can glide across or click on the red dots to get detailed time-zone information.

Figure 1 focuses on a solution in which reference hubs are 2, 10, and 18 h ahead of UTC (e.g., Berlin, Sydney, and Phoenix in the summer). The three boxes show geographic areas within which additional hubs are ±2 h relative to reference hubs. Note that this is only a preliminary sketch; it does not account for changes in DST from summer to winter or from 1 yr to the next, or time-zone differences that do not correspond to whole numbers of hours. Although there are thousands of reasonably possible hub locations in the world (according to internet sources, roughly 500 cities have more than a million inhabitants and 4,000 have more than 100,000), for purpose of argument we will focus on a few arbitrarily selected locations.

The same figure can be looked at in a different way. The boxes illustrate different times of day in a snapshot of the earth’s surface. If it is morning in the left box, it is evening in the middle, and night on the right. To imagine the sun moving during the day, imagine the boxes moving to the left.

If the daily program at *Berlin, Sydney, Phoenix* is confined to 9–13 and 17–21 local time, which additional hubs are appropriate? Tel Aviv is an interesting location, being 1 h ahead of Berlin. There, the program blocks would be 10–14 and 18–22. New York is 2 h ahead of Phoenix, so program blocks there would be 11–15 and 19–23 daily, but Chicago would be preferable, with program blocks 10–14 and 18–22. Beijing is 2 h earlier than Sydney, at least in the Northern summer, with program blocks 7–11 and 15–19. That is possible, but Tokyo would be preferable, with 8–12 and 16–20. Delhi would be rather inconvenient, but still possible, being 2.5 h later than Berlin. Rio de Janeiro would be hardly possible, being 3 h ahead of Phoenix, but Bogotá would be only 1 h ahead, making it an interesting South American location.

Another possible hub combination is *London, Tokyo, Los Angeles* (UTC 1, 9, 17). This applies if the boxes in Figure 1 are shifted 1 h to the left. In this case, New York can hardly be an additional hub, being 3 h ahead of Los Angeles. An East-coast US hub would be better located in Chicago (2 h ahead). Beijing would be 1 h earlier than Tokyo - no problem. There could be an additional central European hub, 1 h later than London, and again Tel Aviv is possible, 2 h later. Delhi would be very inconvenient: 3.5 h earlier than Tokyo, or 4.5 h later than London. Rio de Janeiro would be similarly inconvenient – 4 h later than Los Angeles or 4 h earlier than London. Bogotá would be a better location for a South American hub – 2 h later than Los Angeles.

Consider now the combination *Yerevan, Auckland, New York* (UTC 3, 11, 19). In this case, the boxes in Figure 1 are shifted 1 h to the right. This option is not promising for Los Angeles or London, each being 3 h earlier than the closest hub. Colleagues on the West coast of the USA could travel to an additional hub in Phoenix, 2 h earlier than New York. Colleagues from the UK could travel to a central European hub that is 2 h earlier than Yerevan. Delhi would be comfortable, being 1.5 h later than Yerevan, but Beijing would be a poor location, being 4 h later. Sydney would be 2 h earlier than Auckland.

## Further Organizational Issues

### Technical Setup

The success of a multi-hub conference depends crucially on the reliability of the technology. All sessions must begin and end exactly on time. Technical delays can barely be tolerated. The success of the conference, therefore, depends to a large extent on its technicians and their motivation, creativity. It also depends on their social and intercultural skills in interacting with technicians at other hubs, their willingness to work at unusual times at short notice, and their ability to respond promptly, constructively and creatively when unexpected problems arise.

Each hub needs a head technician (for example, a student in audio engineering), and one of those head technicians will be responsible for the entire conference. He or she will prepare detailed guidelines and work together with the head technicians at the other hubs to ensure that procedures are understood and followed. That includes responding quickly to possible problems (trouble shooting) without delaying the program. In addition, every hub should rehearse independently with every other hub in advance of the conference. At ICMPC15/ESCOM10, there were no notable technical problems and no delays or cancelations for technical reasons. Before the conference, like all conference organizers we were worried that things might go wrong. But we also learned that technical problems can be avoided by sufficient advance practice and patient communication with organizers and technicians at other hubs.

In principle, every video communication service that allows for high quality two-way interaction can be used to connect the conference rooms at different hubs. Ideally, the same service is used to connect virtual active participants. Some of these services also have webinar features, which allow sharing the content with a large number of additional passive virtual participants. If such features are not available, the complete session at one of the hubs can be broadcasted live using a one-way streaming platform. A comprehensive list of tools for virtual conference organizers is available, summarizing both two-way and one-way options (Association for Computing Machinery, 2020).

At ICMPC15/ESCOM10 in 2018, the organizers (including RP and NM-K) optimized audiovisual quality and reliability by simultaneously running one-way and two-way communication software in each room throughout the conference. The one-way communication was an unlisted *YouTube* stream in conjunction with *Open Broadcaster Software (OBS)*, which we used to stream to *YouTube* and juxtapose the talking head of the speaker with the *Powerpoint* image. The two-way communication was *Zoom*. Before each session, a one-way stream was started, which continued throughout the session. Before the start of each talk, there was two-way communication with the speaker. At the start of the talk, the technician switched to a one-way stream, keeping the two-way communication running in the background. The switch changed only what images were projected and what sound was heard (Parncutt et al., 2019). The audience at the remote hub watched the one-way stream. The subsequent discussion was two-way.

This setup takes advantage of the benefits of each system while at the same time reducing the chance of delays. A one-way stream offers high audiovisual quality (with a time delay of a few seconds to a minute), whereas a two-way platform offers quasi-instantaneous communication. If there is a two-way problem, the technician can switch to one-way and ask the audience to write questions in the chat feed. The speaker can then answer acoustically. If the one-way stream fails, the two-way platform provides an immediate backup. In that case, virtual participants viewing the stream will experience an interruption, but if sessions are recorded locally, the missed content can be made available later.

Given our focus on inclusivity, it is important to note that YouTube and other services that might be useful for organizing a multi-hub conference cannot currently be used in China. Levitis et al. (2021, section A.2.1) recommend instead:

HackMD or Microsoft 365 (in place of Google Docs) for collaborative editing, WeChat (in addition to Twitter) for general announcements, DouYu (in addition to YouTube) for live broadcasting, and Bilibili (in addition to YouTube) to archive videos.

Another option uses only two-way communication. This is becoming increasingly relevant as the audio-visual quality of two-way meeting services improves. Software now exists that prioritizes broad-bandwidth and low-latency audio over video transmission and thereby enables near-physical acoustic communication between groups of people at three to four hubs. The audio streaming protocol would need neither strong dynamic compression nor echo cancelling, which when overly used (as they often are in default settings) reduce speech intelligibility. In most conferences, the meaning communicated in paper presentations, panel debates, keynotes, and discussions (Q&A) tends to rely on spoken communication more or less throughout and only occasionally depend on high-quality visual communication (e.g. when showing a movie or motion graphics). Therefore, the streaming protocol needs to allow for dynamic allocation of data bandwidth between audio and video streams for optimal performance. In any case, the physical spaces need to establish good acoustic comfort (i.e. through careful passive acoustic control, microphones, and loudspeakers).

Special attention must be paid to the audio routing. At each hub, the incoming sound from the two-way software is routed to a public address (PA) system in the room. During the discussion period, a wireless, hand-held microphone with a directive pickup pattern is handed to the audience members, which is routed both to the local PA and the outgoing stream. The presenter uses a clip-on (lavalier) microphone. The microphone setup is critical to avoid acoustic feedback when omnidirectional microphones pick up all sound in connected rooms. Also, it is important to route the incoming sound only to the PA system, and not back to the outgoing sound. All in all, the required hardware amounts to at least one handheld, wireless microphone for the audience, one lavalier microphone for the presenter, a sound mixer that allows for the required routing, and a PA system, which is commonly available in conference rooms. Rehearsals between hubs should take place in exactly the same rooms and using exactly the same equipment as during the conference.

Each hub should do some advance research on internet speeds in different rooms of their institution at different times of day, comparing Wi-Fi with cable connections, to avoid unpleasant surprises. If streaming is used, it should be tested well before the conference to make sure that no institutional firewalls block the necessary ports.

An advantage of constant electronic communication between hubs is that the entire proceedings are recorded, adding to the conference documentation. Traditionally, documentation is confined to the program, abstracts, and proceedings. Today’s researchers are increasingly relying on recorded videos to learn about the research of others and interact with each other. Conference videos are also becoming an increasingly important resource for students. After the conference, participants can put their videos in the internet or link them to their homepages, increasing outreach. On online program with video links is the conference’s lasting legacy.

We see promising opportunities for imaginative software engineers with experience of different conference formats to create a new software tool. The tool would offer conference organizers a flexible platform for organizing and presenting multi-hub conferences in a way that is environmentally sensitive and DEI-aware. Currently promising software could be combined and new developments included as they become available. The challenge is not only technical, but also organizational and economical.

### Virtual Socializing and Networking

Replacing the traditional coffee break, virtual socializing and networking is an essential element of virtual, hybrid, and multi-hub conferences. The success of traditional single-location conferences depends to a large extent on social interaction. Organizers of multi-hub conferences will therefore need to study the options and offer diverse, appropriate opportunities to participants.

During a virtual socializing session, any conference participant should have the opportunity to contact any other participant at any location toward the East in AM slots and toward the West in PM slots, either independently or within previously organized meetings. Each hub can have a room devoted to virtual socializing with several computers, each with three sets of headphones and one microphone. People can also use their own mobile phone and headset and use software that also works on a phone. In that way, virtual participants can also join conversations. New online platforms are emerging and developing that offer different advantages; they may need to be systematically evaluated in realistic simulations. Some software support for remote immersive conferencing has been tried, including *Third Space* (th1rdspac3.com) and *Gather* (gather.town), which might offer a richer experience.

Specific virtual socializing events can be organized in advance and included in the program:

• focus groups: discussions addressing specific topics that anyone can join spontaneously
• new supervisions: pre-arranged meetings between senior and junior participants
• reunions: pre-arranged meetings between participants who already know each other
• free sessions: random meetings in groups of any size

The discussions that follow each talk are also a form of virtual socializing. To encourage maximum participation, it can help to offer various ways of contributing (speaking with or without video, writing a question, using a mobile phone or laptop).

At each hub, a student assistant can be put in charge of virtual socializing. Across hubs, virtual socializing assistants can meet virtually before the conference for planning purposes. All conference participants may be asked in advance which social events they would like to attend and whether they are happy to provide confidential information that will help them to be matched up with colleagues of similar interests. In our experience, structured virtual socializing sessions need to be planned in advance, and should involve specific named participants, otherwise the opportunity will be missed.

From a technological point of view, there is great potential for making virtual socializing sessions more engaging. In the future, virtual reality / augmented reality technology (VR/AR) might be key contributors. Le et al. (2020) conducted a hybrid conference using social virtual environments, which allow avatar-based interaction, optionally using a head-mounted display. Participants were more likely to socialize with others when using the social VR platform as opposed to a one-way streaming option. Social VR environments are constantly improving, and together with augmented reality communication, they could form the basis of more natural virtual socializing sessions.

Virtual poster sessions are another opportunity to socialize while at the same time getting to know new research. Again, they can be organized in various ways that require advance planning. Organizers will need to explore the current electronic options for poster presentation, and poster presenters need to be informed well in advance about how such sessions will work. A currently promising platform is *SpatialChat* (spatial.chat).

### Financial Issues

The budget at each hub of a multi-hub conference is similar to the budget for a regular conference at that location, based on the number of physically present participants. It includes room rent, food and refreshments (including reception and banquet, if applicable), printing, conference bags and other giveaways such as cups, and bank fees. Equipment costs are low if the hub is hosted by a university that makes available the necessary computers, data projectors, loudspeakers, and cable and wireless internet connections. Registration fees are cheaper in countries with lower GDP.

Costs are higher at the organizing hub, which organizes abstract submission and peer review on behalf of all hubs. For this purpose, a conference manager may be employed for several months before and during the conference – comparable with a conventional single-location conference.

Relative to a conventional conference, the main expense at the non-organizing hubs is wages for the local head technician. That person might work for a month (starting 3 wk before the conference) to ensure that all hardware and software operates properly, checking everything together with the conference’s global head technician. One or more additional technicians may be needed at each hub for the duration of the conference – one for each conference room in which live or virtual talks are held. Their wages can be covered in part by charging a small fee to virtual participants.

Some universities are charging high fees for room rent for large lecture theatres – those which are suitable for large international conferences. In a multi-hub conference, the hubs are smaller than a conventional single-location conference, so hub organizers will need to cater for a smaller number of participants. For that purpose, regular smaller teaching rooms may suffice, for which little or no rent may be payable.

### Other Organizational Issues

*Legal issues.* An important decision to be made in advance is whether conference materials will be publicly available or whether they will be limited to paid-up and registered participants. In the latter case, internet security is never 100%. Nor is it reasonably possible to trust all conference participants to avoid putting links to conference materials in the internet. Therefore, all speakers at such a conference should present as if their talk were public. They should carefully avoid creating legal problems in areas such as copyright or defamation, especially as some streaming platforms automatically terminate streams when registered, protected material is broadcasted.

*Accommodation.* At a multi-hub conference, sessions may start earlier and/or end later than usual. Hub organizers need to ensure that there is accommodation in the usual categories at walking distance from the conference location. If public transport is necessary, check that it is running at the required times.

## Discussion

We have described a conference format that solves several of the problems emerging from current formats as they are adapted in response to two global crises: the COVID-19 epidemic and climate change. Our main proposal is to establish three reference hubs that are exactly 8 h apart, before considering other possible hubs. Our recommendations are based primarily on our practical experience as conference organizers and motivated by our belief in the importance of reducing emissions and improving inclusion. In that sense, our contribution lies between an opinion paper and evidence-based research. The success of our proposals will depend on how they are implemented by different academic societies, including our own, in coming years.

The task of finding the best conference format under constraints of low emissions and high DEI, and considering the current state of audiovisual technology and associated human limitations, may be regarded as a logistic optimization problem. We are trying to maximize a set of complex, interesting, intuitively understood variables, such as environmental friendliness, DEI, academic knowledge generation, group creativity, and the “conference experience.” At the same time, we are trying to minimize flying distances and time-zone differences. The solution that we have presented has been constructed by a process of informed trial and error in authentic high-level academic conference situations, in which the authors were both organizers and participants. It may be possible to carry out the optimization process more systematically than we have done.

Regardless of how technologies for multisensorial communication over large distances develop, international conferencing will need to establish reliable formats to handle time-zone differences within an equitable and sustainable model. Our recommendations are tentative, given that a conference that fully exploits the advantages of a multi-hub type has yet to take place. Based on our experience, we predict that the presented model will be successful and sustainable. Once a given academic community has implemented our proposal and reassured itself that the technology is reliable, acceptance among participating colleagues will be high, and similar formats will be planned for further conferences.

We believe that our recommended format is appropriate for any academic or industry-based discipline, whether humanities, sciences, or practically oriented. Whenever expert colleagues come together to share and discuss the current directions of their discipline, a conference in this format is promising. A similar format could be used for politics, business, hobbies, or cultural events. For example, the first author is involved in a local choral festival (Voices of Spirit, Graz) that is planning a global, multi-hub event involving choirs from many different countries (“Voices for Future”).

Relative to all global CO_2_ emissions, the contribution of academic conferences is small: roughly 7 million tons of carbon per year, or less than 0.1% of global fossil fuel consumption (Parncutt and Seither-Preisler, 2019). From that perspective, the proposed reforms have a poor cost-benefit ratio. But low-carbon conferences also allow academics to drastically reduce their personal carbon footprints and, in that way, to act as role models for other people in their sphere of influence. They also allow academic conferences as a whole to drastically reduce their emissions, acting as models for conferences in business and politics. Academics’ networking, outreach, and trained ability to understand and explain complex issues allow them to relatively easily change their travel behaviors and in so doing to inspire others to follow their lead (cf. Anderson, 2013; Waring et al., 2014; Fraser et al., 2017; Glover et al., 2018; Grant, 2018; Middleton, 2019; Middleton, 2021; Quinton, 2020; van Ewijk and Hoekman, 2020; Levitis et al., 2021).

There is also an interesting moral issue about the necessity of unilateral action as a step toward multilateral action. If everyone waits for others to take the first step, nothing happens. The best solution may be a compromise between unilateralism and multilateralism called *minilateralism* (Eckersley, 2012). A conference organizer who follows the present proposals and guidelines is arguably applying this principle in the context of a growing trend toward low-carbon conference formats.

We have not addressed all relevant issues. Language is an interesting one. The easiest option for most academic societies is to insist that everything happens in one language. Most often, that language is English, although there are interesting exceptions (e.g., a global conference on Spanish literature). Whereas a monolingual approach discriminates against speakers of other languages, it avoids a potentially worse form of discrimination, namely the smaller audiences that are attracted to talks by speakers of minority languages. The negative consequences of insisting on one *lingua franca* can be reduced by encouraging participants to give each other linguistic support. Such exchanges can be organized centrally – an additional means of helping participants make new social and academic contacts.

Recent technical advances are making it increasingly possible for multiple languages to be included in one conference on the same level. However, automatic real-time transcription and translation is not yet good enough for most conferences due to their discipline-specific technical jargons (even if translation systems are trained in advance). If prerecorded videos are used, captions can be added, making it possible for the presenter to speak in one language while the captions explain in another. Levitis et al. (2021) recommend adding “crowd-sourced captioning in multiple languages.”

Skeptics may object that our proposal inhibits a tried and tested approach to collective academic creativity, by preventing (or at least discouraging) colleagues from flying halfway across the globe. But our format also allows many colleagues to participate for the first time, without discrimination. At ICMPC15/ESCOM10 in 2018, participant satisfaction was highest at the Argentinian hub for this reason. This comparison suggestions that, if we focus on the long-term creative academic outputs that arise indirectly from international conferences, the advantages of our proposal outweigh the disadvantages.

Another possible objection involves the lack of sessions that include all conference hubs. That is a result of international time zone differences, which represent another hard physical constraint. We propose limiting all global meetings to about 2/3 of all hubs. In so doing we are assuming that most colleagues prefer not to radically disrupt their sleeping habits during conferences. The success of a conference depends on participants being awake and well-rested, which in turn means limiting working hours in a reasonable and familiar way – consistent with more general concerns about work-life balance in academe (Siddiquee et al., 2016). The psychological state of conference participants that are immersed in current issues specific to their academic discipline and striving for high academic standards can be compared to flow (Ljubin-Golub et al., 2018). To promote or maintain flow, whether in face-to-face or virtual interaction (Ghani et al., 1991), the challenges of the conference should not exceed the abilities of the participants. Sleep deprivation should be avoided because it affects cognitive performance (Alhola and Polo-Kantola, 2007), which in turn affects both enjoyment and long-term academic outcomes. If participants lose sleep near the start of a conference, the result might be missed sessions or poor working atmosphere later on.

Most of the socializing at a multi-hub conference occurs among the physically present at each hub. As often as reasonably possible, that interaction should be mixed with virtual presentations and virtual socializing. Given that the best feeling of conference community is created face-to-face, to what extent can virtual communication replace it? This question, we believe, cannot be separated from issues of climate change and inclusion. Mounting scientific evidence about the urgency of mitigating climate change suggests that mitigation is more important than maintaining the traditional conference experience. At the same time, there is growing awareness that colleagues should be included in conferences who would otherwise have been excluded due to lack of financial resources, location, disability, or caring commitments. Our proposed format means that many colleagues will participate who would not otherwise have done so. In this sense, our proposed solution is an optimal but tentative compromise. It could be improved by developing alternative strategies to promote global community. For example, audiences from different geographical areas can be mixed within the same talks, sessions, meetings, and other events; and new communication technologies can be explored and tried out to make virtual communication seem increasingly real.

Finally, body language plays an important role in face-to-face communication (Ferrara and Hodge, 2018). In virtual and semi-virtual interaction, important signals may be missing or misinterpreted. Relevant situations include the following. In virtual presentations at hybrid and multi-hub conferences, local audiences discuss content in two ways: face-to-face interaction and virtual interaction with one or more remote audiences. All active participants use cameras (unless bandwidth is to low that they need to turn off their camera). Each session has a chair who is responsible for managing questions and a technician who is responsible for managing the sound.

Whereas virtual communication may never have the same quality as face-to-face, technological improvements in coming years and decades will gradually close the gap. At a 30-min talk timeslot at a multi-hub conference, the speaker might speak uninterrupted for 20 min followed by 7 min of questions after which there is a short break before the next talk. During the talk proper, the speaker’s attention will be focused on the local audience, although one or more virtual audiences will also be watching. The speaker will respond to visual or auditory cues from the local audience (expressions, coughing), but not from the virtual audience. If there is feedback of this kind from the virtual audience, it may be misinterpreted. The question session works differently: feedback from different sources is managed by the session chair, who gives turns to local and virtual audiences. This is a skill that needs to be learned, and session chairs may have little experience; but as technologies improve and people become used to interacting with them, the problems will diminish.

To conclude, although very many combinations of conference hubs are theoretically possible, the specific constraint that we have proposed – three temporally equally spaced reference hubs – appears to be optimal in the sense that it maximizes program sharing and hence global academic exchange.
